# Supplementary material for: Preconception risk factors and health care needs of pregnancy-planning women and men with a lifetime history or current mental illness: A nationwide survey
Source: PLoS One. 2022 Jun 22;17(6):e0270158. doi: 10.1371/journal.pone.0270158 (PMC9216596; doi:10.1371/journal.pone.0270158)
Supplement: Attachment — Submitted filename: PLoS One_response to reviewers 2.docx [file pone.0270158.s003.docx]

**Response to the Editor:**

1. Please address reviewer 2's minor points.

- We have done so, with responses below.

**Response to Reviewer 1:**

1. I greatly appreciate the author's responses and care in addressing them all in the manuscript or within the author replies.

- Thank you for your positive assessment of our revision.

**Response to Reviewer 2:**

1. Response rate was not addressed because collection methods did not allow for this data collection, this should be added to a limitations of the study

- We have added this as a limitation. See Lines 385-386: “We did not have information on response rate, as this was an online survey; we only had the number of completed responses.”

2. Table 2 is very busy with lots of data - I would move the headings to the center and highlight each subsection to make following the data easier for readers

- We have made this change.

3. Line 376 has inaccurate punctuation

- We have edited the punctuation, as follows: “Further, while the number of men was small compared to women (which did not allow for stratified analysis), we were able to calculate sex-adjusted estimates that can be used to guide future research focused on men specifically.”
